# An Oxygen-Sensing Two-Component System in the *Burkholderia cepacia* Complex Regulates Biofilm, Intracellular Invasion, and Pathogenicity

**DOI:** 10.1371/journal.ppat.1006116

**Published:** 2017-01-03

**Authors:** Matthew M. Schaefers, Tiffany L. Liao, Nicole M. Boisvert, Damien Roux, Deborah Yoder-Himes, Gregory P. Priebe

**Affiliations:** 1 Division of Critical Care Medicine, Department of Anesthesiology, Perioperative and Pain Medicine, Boston Children’s Hospital, Boston, Massachusetts, United States of America; 2 Department of Anaesthesia, Harvard Medical School, Boston, Massachusetts, United States of America; 3 IAME, UMR 1137, INSERM, Université Paris Diderot, Sorbonne Paris Cité, Paris, France; Service de Réanimation médico-chirurgicale, Hôpital Louis Mourier, AP-HP, Colombes, France; 4 Department of Biology, University of Louisville, Louisville, Kentucky, United States of America; University of Washington, UNITED STATES

## Abstract

*Burkholderia dolosa* is a member of the *Burkholderia cepacia* complex (BCC), which is a group of bacteria that cause chronic lung infection in patients with cystic fibrosis (CF) and can be associated with outbreaks carrying high morbidity and mortality. While investigating the genomic diversity of *B*. *dolosa* strains collected from an outbreak among CF patients, we previously identified *fixL* as a gene showing signs of strong positive selection. This gene has homology to *fixL* of the rhizobial FixL/FixJ two-component system. The goals of this study were to determine the functions of FixLJ and their role in virulence in *B*. *dolosa*. We generated a *fixLJ* deletion mutant and complemented controls in *B*. *dolosa* strain AU0158. Using a *fixK-lacZ* reporter we found that FixLJ was activated in low oxygen in multiple BCC species. In a murine pneumonia model, the *B*. *dolosa fixLJ* deletion mutant was cleared faster from the lungs and spleen than wild-type *B*. *dolosa* strain AU0158 at 7 days post infection. Interestingly, the *fixLJ* deletion mutant made more biofilm, albeit with altered structure, but was less motile than strain AU0158. Using RNA-seq with *in vitro* grown bacteria, we found ~11% of the genome was differentially expressed in the *fixLJ* deletion mutant relative to strain AU0158. Multiple flagella-associated genes were down-regulated in the *fixLJ* deletion mutant, so we also evaluated virulence of a *fliC* deletion mutant, which lacks a flagellum. We saw no difference in the ability of the *fliC* deletion mutant to persist in the murine model relative to strain AU0158, suggesting factors other than flagella caused the phenotype of decreased persistence. We found the *fixLJ* deletion mutant to be less invasive in human lung epithelial and macrophage-like cells. In conclusion, *B*. *dolosa fixLJ* is a global regulator that controls biofilm formation, motility, intracellular invasion/persistence, and virulence.

## Author Summary

In people with cystic fibrosis (CF), infection with bacteria in the *Burkholderia cepacia* complex (BCC) is often associated with clinical deterioration. In a whole-genome sequencing study of the BCC species *B*. *dolosa*, we previously identified the *fixL* gene of the FixL/FixJ two-component system called FixLJ to be under strong positive selective pressure during chronic infection. In this study we show that low oxygen levels activate FixLJ, and that a mutant of *B*. *dolosa* in which the *fixLJ* genes are deleted is less able to persist in the lungs and spread to the spleen in a lung infection model in mice. The *fixLJ* deletion mutant has defective motility and intracellular survival within epithelial cells and macrophage cell lines. However, a flagella mutant is fully infectious, suggesting that low motility is not responsible for the *fixLJ* deletion mutant’s inability to persist within the host. Analysis of global RNA expression shows that the *fixLJ* system regulates many genes, indicating that multiple pathways likely contribute to the low virulence of the *fixLJ* deletion mutant. In conclusion, *B*. *dolosa* FixLJ compose an oxygen sensor that regulates the ability of the bacteria to survive inside host cells.

## Introduction

*Burkholderia dolosa* is a member of the *Burkholderia cepacia* complex (BCC), which is a group of related Gram-negative bacilli that can be dangerous respiratory pathogens for patients with cystic fibrosis (CF) [1, 2]. BCC can also cause outbreaks of bacteremia or respiratory infection in hospitalized non-CF patients, including the recently discovered cluster of cases of BCC respiratory infection linked to contaminated stool softener (docusate) [3]. BCC are also common pathogens for individuals with chronic granulomatous disease [4]. Among CF patients in the United States colonized with BCC, the species most commonly seen are *B*. *cenocepacia* (45%), *B*. *multivorans* (35%), *B*. *vietnamiensis* (6%), *B*. *cepacia* (6%), and *B*. *dolosa* (3%), although there is significant variability based on geographic region and institution [5]. A number of studies have shown an association between infection with BCC and clinical pulmonary deterioration of CF patients [6–10]. The so-called “*cepacia* syndrome” refers to the clinical presentation of fevers, leukocytosis, elevated erythrocyte sedimentation rate (ESR), and progressive severe pneumonia, sometimes with bacteremia, in CF patients occurring relatively soon after acquiring BCC, with a mortality rate initially reported at 62% [11]. BCC infection in CF patients can be transmitted person-to-person, and multiple outbreaks have been described, including one of a highly antibiotic resistant strain of the BCC species *B*. *dolosa* among almost 40 CF patients at Boston Children’s Hospital [12]. This outbreak has been associated with accelerated decline of lung function and decreased survival [13]. BCC in general are commonly multidrug resistant (MDR) or extensively drug resistant (XDR) pathogens, highlighting the necessity for novel approaches for treatment of these bacteria [14].

**Fig 1:**
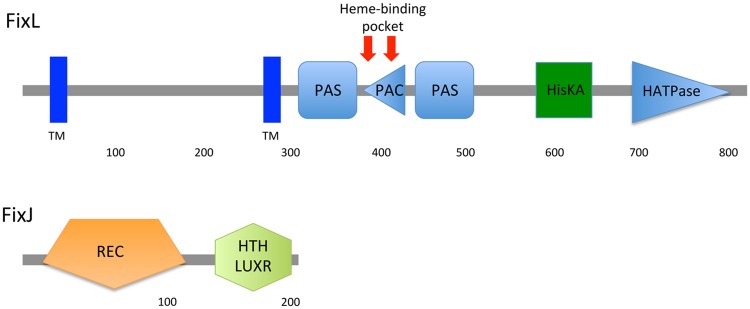
Predicted domains of *B*. *dolosa* strain AU0158 FixL and FixJ. Domains predicted by SMART [25]. PAS domains (named after 3 proteins in which they occur, namely, Per, Arnt, and Sim) are seen commonly in signaling proteins where they function as signal sensor and often include a cofactor such as heme. Domain abbreviations: TM- transmembrane, PAC- Motif C-terminal to PAS motif, HisKA-histidine kinase, HATPase- histidine kinase-associated ATPase, REC-CheY homologous receiver domain, and HTH LuxR-helix-turn-helix-Lux regulon (DNA binding domain).

In previous work, we sequenced the genomes of 112 *B*. *dolosa* isolates collected from 14 individuals over 16 years from the Boston Children’s Hospital outbreak [15]. We found that a subset of genes having 3 or more point mutations contained 18 times as many non-synonymous mutations than expected by neutral drift and were thus under strong positive selection (dN/dS = 18, 95% CI: 4.9–152.7) [15]. Positive selection here refers to the type of mutations that were seen (non-synonymous versus synonymous), not mutations that necessarily make a protein more active. One of these genes (BDAG_01161, AK34_969), which had a remarkable 17 non-synonymous mutations, encodes a protein with homology to FixL of the FixLJ two-component system of *Sinorhizobium* and *Caulobacter*. FixL in those species is a sensory histidine kinase that detects oxygen tension and phosphorylates the transcription factor FixJ under low oxygen conditions, which subsequently induces transcription of *fixK* [16]. The predicted domains of *B*. *dolosa* FixL and FixJ (BDAG_01160, AK34_968) are depicted in Fig 1. Both proteins have domains consistent with their putative function as a two-component system [17]. FixL is predicted to contain PAS domains and a heme-binding pocket where oxygen binding to FixL likely occurs [16]. Notably, *B*. *dolosa fixK* (BDAG_04180, AK34_4936) has a *B*. *cenocepacia* homolog (BCAM0049) that was found to be up-regulated during growth in a low-oxygen environment in two separate studies [18, 19]. A high number of non-synonymous mutations in the *B*. *multivorans fixL* homolog (BMD20_10585) was recently reported in another whole-genome sequencing study of isolates recovered from chronically infected CF-patients [20], suggesting that the FixLJ system has a similar role in all 3 BCC species commonly seen in CF patients. The FixLJ system in *Rhizobium* and *Caulobacter* induce expression of genes required for fixing of atmospheric nitrogen in the absence of oxygen [21]. While *B*. *vietnamiensis* and *Burkholderia* species outside of the BCC can fix nitrogen, most members of the BCC, including *B*. *cenocepacia*, *B*. *multivorans*, and *B*. *dolosa* lack the genes necessary for nitrogen fixation [22–24].

In this study, we sought to determine the functions of *B*. *dolosa* FixLJ and their role in pathogenesis. We hypothesized that the FixLJ system senses oxygen depletion and regulates genes involved in virulence based on its function in other species. We show that *B*. *dolosa* lacking *fixLJ* are unable to induce transcription of a *fixK-lacZ* reporter grown in low oxygen, have altered expression of ~11% of the genome, and are cleared faster in a murine lung infection model. Interestingly, the *fixLJ* deletion mutant is less motile and less invasive but makes more biofilm than the parental strain. Non-motile *B*. *dolosa* lacking flagella show no defect in the murine infection model at comparable dosages, indicating that a reduction in flagella expression is not the mechanism for decreased persistence seen in the *fixLJ* deletion mutant. Overall, the *fixLJ* system regulates a large number of genes and is critical for *B*. *dolosa* pathogenicity.

## Results

### FixLJ induces transcription of *fixK* in response to low oxygen

To determine the stimulus that induces the *fixLJ* pathway, we constructed *B*. *dolosa fixK* promoter-driven LacZ reporter plasmid using the pSCrhaB2 backbone [26]. *B*. *dolosa fixK* (AK34_4936) is the homolog of *S*. *meliloti fixK*, which is a target of the *fix* pathway [16].

**Fig 2:**
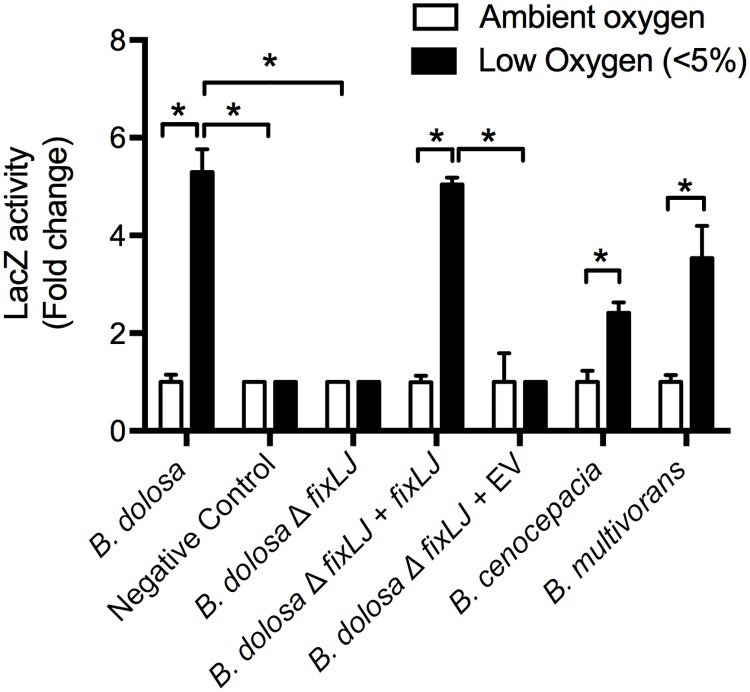
*Burkholderia* FixLJ functions as an oxygen sensor. *B*. *dolosa* (strain AU0158 or its *fixLJ* deletion mutant), *B*. *cenocepacia* (strain J2315), and *B*. *multivorans* (strain ATCC23344) carrying a *pfixK-lacZ* reporter plasmid. Negative Control denotes reporter plasmid carrying *S*. *meliloti fixK* promoter sequence. EV denotes empty complementation vector. LacZ activity was quantified by Miller Units. Bars represent the means of triplicate biological replicates and error bars represent one standard deviation (representative of three independent experiments). *P<0.001 by 1-way ANOVA with Tukey’s multiple comparison test.

We conjugated this plasmid into the BCC strains and measured activation of the pathway when grown in low oxygen (<5%) relative to growth in ambient oxygen. There was a ~5 fold induction in the *fix* pathway (measured by *fixK*-driven activity of LacZ) when grown in low oxygen (Fig 2). This induction was specific for the *B*. *dolosa fixK* promoter sequence as it was lost when using a reporter plasmid containing the *S*. *meliloti fixK* promoter sequence to drive LacZ expression (which served as a negative control). The *fixLJ* deletion mutant was unable to induce *fix* activity in response to low oxygen. When the *fixLJ* deletion mutant was complemented chromosomally with *fixLJ* under the control of its own promoter, induction of *fix* activity in response to low oxygen was restored, demonstrating that induction of the pathway was *fixLJ-*specific. We found that the *fixLJ* deletion mutant has a mild (~7.5 fold) growth defect when grown in ambient and low (<5%) oxygen relative to strain AU0158 (S1 Fig). The *fixLJ* DNA sequences in *B*. *dolosa* (strain AU0158), *B*. *cenocepacia* (strains J2315 & K56-2), and *B*. *multivorans* (strain ATCC17616) are nearly identical (94–95% identity), suggesting FixLJ has the same function in all species. We conjugated the *B*. *dolosa fixK* reporter plasmid into *B*. *cenocepacia* (strain J2315) and *B*. *multivorans* (strain ATCC17616) and found the same induction in response to low oxygen, demonstrating this pathway functions similarly in these two more commonly encountered BCC species.

### *fixLJ* deletion mutants are cleared faster in a murine pneumonia model

**Fig 3:**
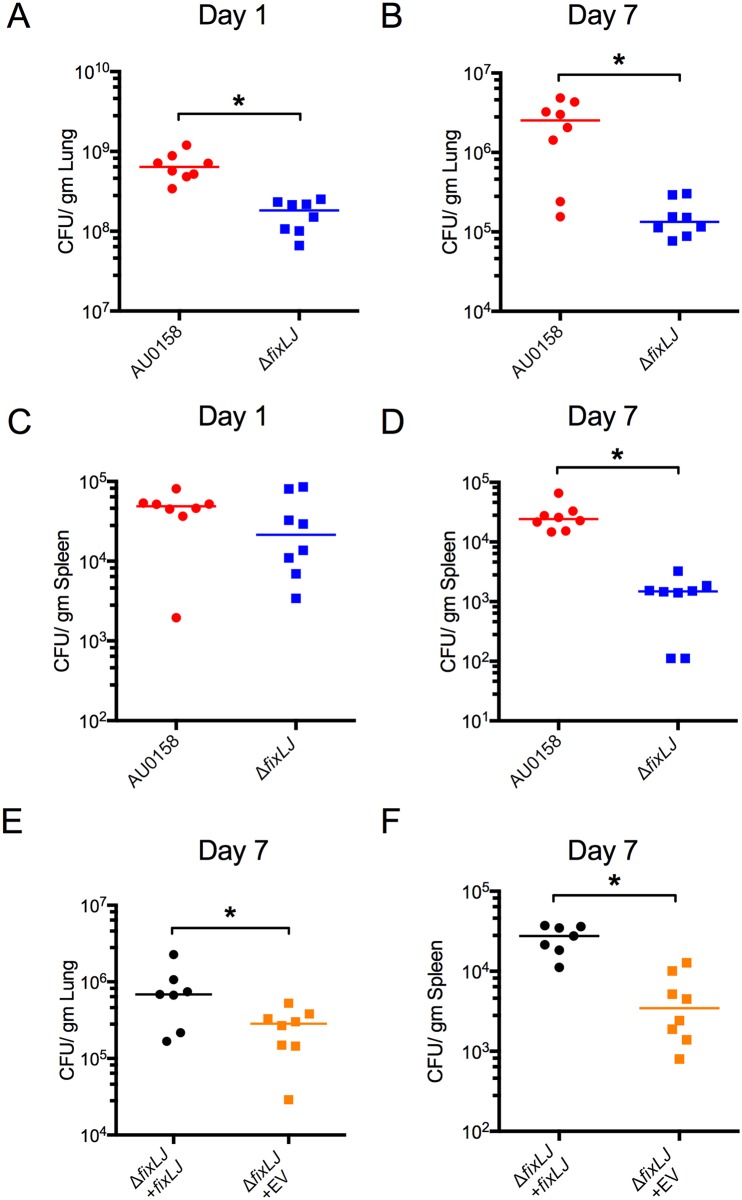
The *B*. *dolosa fixLJ* deletion mutant is cleared faster in a murine pneumonia model. (A-D) C57BL/6 mice were intranasally challenged with ~4x10^8^ CFU/mouse of *B*. *dolosa* strain AU0158 or its *fixLJ* deletion mutant. Bacterial loads were measured at the following sites and time points: (A) Lungs, 1 day after infection; (B) Lungs, 7 days after infection; (C) Spleen, 1 day after infection; (D) Spleen, 7 days after infection. Two *fixLJ* deletion-infected mice had undetectable bacterial levels in the spleen at day 7 (panel D, shown as 10^2^ CFU/gm). Data is representative from 2 separate experiments with 7–8 mice per group. (E-F) C57BL/6 mice were intranasally challenged with *B*. *dolosa* AU0158 *ΔfixLJ* + *fixLJ* (6.7x10^8^ CFU/mouse) or *B*. *dolosa* AU0158 *ΔfixLJ* + empty vector (EV) (7.4x10^8^ CFU/mouse). Bacterial loads were measured 7 days after infection in the lungs (E) and spleen (F). Data are derived from one experiment done with 7–8 mice per group. Each point represents one mouse, and bars represent medians. *P<0.05 by Mann Whitney U test.

To determine the role of the *B*. *dolosa fixLJ* system in virulence, we infected C57BL/6 mice with ~4x10^8^ CFU/mouse of *B*. *dolosa* strain AU0158 or its *fixLJ* deletion mutant and measured the bacterial load within the lungs and the spleen at 1 and 7 days post infection. As early as 1 day after infection, there was a significant three-fold reduction in the amount of bacteria recovered from the lungs of mice infected with the *fixLJ* deletion mutant compared to mice infected with the wild-type strain AU0158 (Fig 3A). At 7 days, there was a 18-fold reduction of viable counts of the *fixLJ* deletion mutant compared to strain AU0158 (Fig 3B). One day after infection there was no significant difference in the amount of bacteria recovered from the spleens of mice infected with AU0158 or the *fixLJ* deletion mutant (Fig 3C), suggesting there is no defect in the ability of the *fixLJ* deletion mutant to disseminate into the spleen. Seven days after infection there was a significant, 16-fold reduction in the number of bacteria recovered from the spleen of mice infected with the *fixLJ* deletion mutant relative to strain AU0158 (Fig 3D). Mice infected with the *fixLJ* deletion mutant chromosomally complemented with *fixLJ* under the control of its own promoter had significantly higher bacterial loads within the lungs (Fig 3E) and spleen (Fig 3F) at 7 days post infection compared to mice infected in *fixLJ* deletion mutant chromosomally complemented with an empty vector, demonstrating that this reduction of *in vivo* fitness is *fixLJ*-specific. To ensure that the bacteria are causing an infection and not merely colonizing the lungs, we examined histopathology of the lungs at day 7 after infection. Mice infected with either strain AU0158 or the *fixLJ* deletion mutant showed signs of inflammation consistent with bacterial pneumonia (S2 Fig). Slides were score by a rodent pathologist who saw no difference in severity of lung inflammation between infection groups.

### *B*. *dolosa fixLJ* deletion mutants produce more biofilm but with a different structure

**Fig 4:**
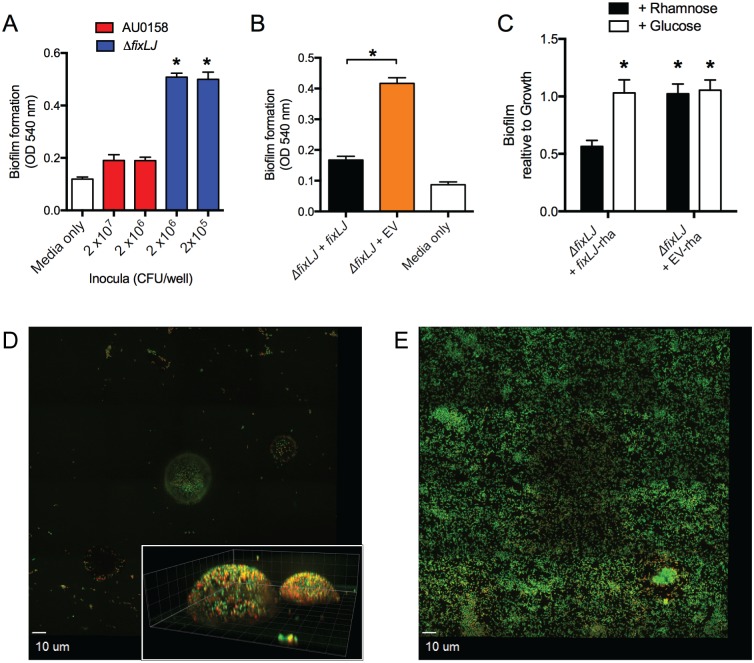
The *B*. *dolosa fixLJ* deletion mutant produces more biofilm by crystal violet staining and has a different biofilm structure. Biofilm formation of *B*. *dolosa* AU0158 constructs on PVC plates as measured by crystal violet staining at 48 hours. (A) *B*. *dolosa* strain AU0158 produces less biofilm than its *fixLJ* deletion mutant. Strains were grown in TSB with 1% glucose at varying inocula. (B) The *B*. *dolosa fixLJ* deletion mutant complemented with *fixLJ* under the control of its own promoter produces less biofilm compared to the strain carrying an empty vector (EV). (C) A *B*. *dolosa fixLJ* deletion mutant complemented with *fixLJ* under the control of a rhamnose-inducible promoter or empty vector grown in LB in the presence of glucose (0.4%, which represses the promoter) or rhamnose (0.4%) and compared to the Δ*fixLJ* + *fixLJ* strain grown in rhamnose-containing medium. For panels A-C, bars represent mean measurements of 5–6 replicates and error bars represent one standard deviation (representative of three independent experiments). *P<0.05 compared to AU0158 by 1-way ANOVA with Tukey’s multiple comparison test. Representative mosaic images of biofilms of strain AU0158 (D) and its *fixLJ* deletion mutant (E) grown on 8-well chamber slides for 48 hours, stained with live/dead stain, and imaged by confocal microscopy. For panels D and E, live bacteria are stained green and dead are red. The inset of panel D shows Z-stack images taken at 1 μm intervals; gridlines denote 5 μm lengths. Images are representative of two independent experiments conducted with four replicates.

To better understand the mechanism of reduced persistence seen in the *fix* deletion mutant, we compared the phenotype of the *fixLJ* deletion mutant to the parental strain in the ability to produce biofilm on PVC plates after 48 hours of growth. Interestingly, we found that the *B*. *dolosa fixLJ* deletion mutant forms significantly more biofilm than the wild-type strain AU0158 (Fig 4A). This phenotype was quite drastic as we are able to see approximately 3-fold more biofilm in wells that were inoculated with 100-fold fewer *fixLJ* deletion mutant CFU compared to *B*. *dolosa* AU0158 (2x10^5^ vs. 2x10^7^ CFU/well). Since the *fixLJ* deletion has the mild growth defect (S1 Fig), the magnitude of the increase in biofilm formation becomes much larger when accounting for bacteria growth (S3 Fig). The *fixLJ* deletion mutant complemented with *fixLJ* under the control of its own promoter formed significantly less biofilm than the *fixLJ* deletion mutant carrying an empty vector (Fig 4B), demonstrating this phenotype is *fix-*specific. We also chromosomally complemented the *fixLJ* deletion mutant under the control of a rhamnose-inducible promoter. When grown in the presence of rhamnose, expression of genes controlled by this promoter are induced, while growth in glucose inhibits expression of genes controlled by this promoter [26]. The rhamnose-inducible complementation of *fixLJ* was able to produce significantly more biofilm when grown in glucose compared to growth in rhamnose (Fig 4C). The *fixLJ* deletion mutant complemented with the empty vector containing the rhamnose-inducible promoter produced significantly more biofilm in both rhamnose and glucose containing media. Here, we determined biofilm measurements relative to the amount of bacterial growth (O.D._600_) since the addition of glucose/rhamnose had differing effects on bacterial growth. We analyzed the biofilm structure grown on glass slides stained with a live/dead stain by confocal fluorescent microscopy. After 48 hours of growth, strain AU0158 had drastically fewer bacteria adherent to the slide, and those that were adherent were in dome-like structures (Fig 4D). The dome-like structures of strain AU0158 had heights of 10–15 μm (Fig 4D, inset), while the *fixLJ* deletion mutant adhered to the glass evenly across the entire surface in a nearly single-cell layer 2–3 μm high (Fig 4E). To better understand the mechanism of biofilm formation and the components of biofilms in strain AU0158 and the *fixLJ* deletion mutant, we treated established biofilm with DNase I, proteinase K, or dispersin B (which cleaves the biofilm polysaccharide PNAG)[27]. We found that strain AU0158 biofilms were susceptible to treatment with any of the three enzymes while biofilms formed by the *fixLJ* deletion mutant were only susceptible to treatment with proteinase K (S3C Fig).

### *B*. *dolosa fixLJ* regulates motility

**Fig 5:**
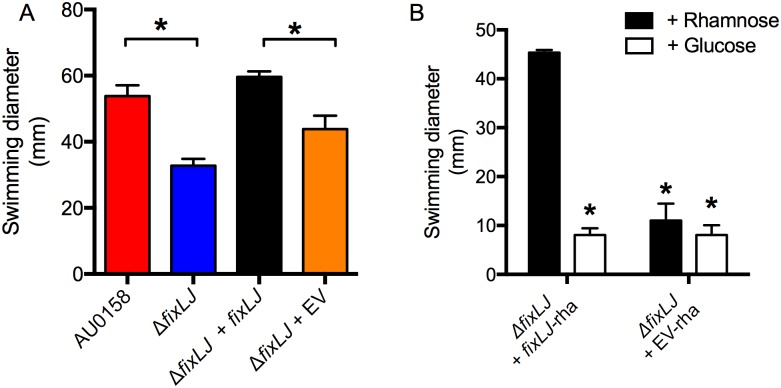
The *B*. *dolosa fixLJ* deletion mutant is less motile. (A) *B*. *dolosa* strain AU0158, its *fixLJ* deletion mutant, and the *fixLJ* deletion mutant complemented with *fixLJ* under the control of its own promoter or empty vector (EV) were plated on low-density (0.3%) LB agar and swimming distance was measured after incubation for 48 hours. *P<0.05 by 1-way ANOVA with Tukey’s multiple comparison test. (B) The *B*. *dolosa fixLJ* -deletion mutant complemented with *fixLJ* under the control of a rhamnose-inducible promoter or empty vector grown in the presence of glucose (0.4%, which represses *fixLJ* expression) or rhamnose (0.4%). *P<0.05 by 1-way ANOVA with Tukey’s multiple comparison test relative to the complemented strain (Δ*fixLJ* + *fixLJ*) grown in rhamnose-containing media. For all panels, bars represent mean measurements of 3–4 replicates and error bars represent one standard deviation (representative of three independent experiments).

Since swimming motility has been correlated with virulence in *B*. *cenocepacia* [28], we next assessed the impact of *fixLJ* deletion on swimming motility. We plated overnight cultures of various *B*. *dolosa* constructs onto low density (0.3%) agar plates and measured the swimming diameter after 48 hours. The *fixLJ* deletion mutant had significantly lower swimming motility compared to AU0158 (Fig 5A). Full motility was restored in the *fixLJ* deletion mutant complemented with *fixLJ* under the control of its own promoter. Using the above-described rhamnose-inducible system to complement the *fixLJ* deletion, we also found that motility was restored when bacteria were grown in the presence of rhamnose but not glucose (Fig 5B).

### The *fixLJ* pathway regulates gene expression

**Fig 6:**
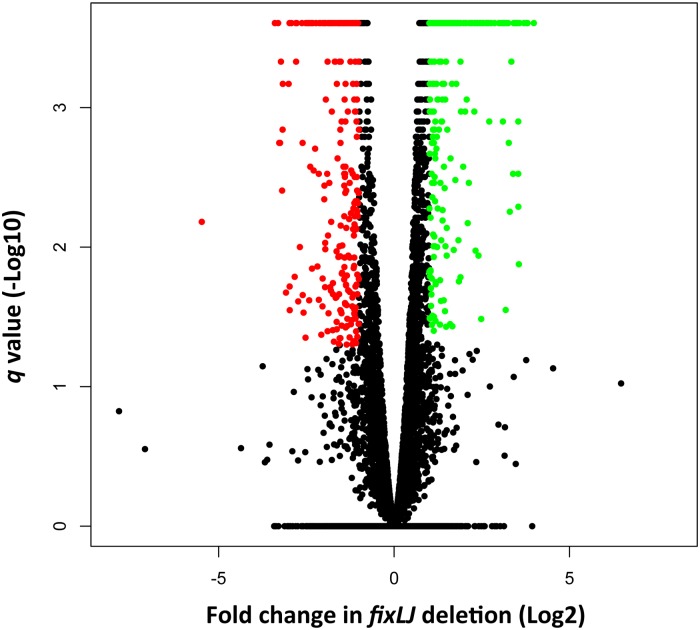
The *fixLJ* pathway is a global regulator of gene expression. Volcano plot depicting the differential regulation of genes in the *fixLJ* deletion mutant relative to the wild-type strain AU0158 measured by RNA-seq. Green dots signify genes with expression 2-fold higher in the *fixLJ* deletion mutant relative to strain AU0158 with a q < 0.05. Red dots signify genes with expression 2-fold lower in the *fixLJ* deletion mutant relative to strain AU0158 with a q < 0.05.

In order to determine the mechanism(s) that are responsible for the decreased persistence *in vivo*, increased biofilm, and decreased motility seen in the *fixLJ* deletion mutant, we compared the transcriptomes of the *fixLJ* deletion mutant to the parental strain AU0158 using RNA-seq. Differentially expressed genes were identified to have at least a 2-fold change (log_2_ >1) and a *q* value <0.05 (Fig 6). Of the 5717 genes in the annotated genome of strain AU0158, 300 genes were significantly up- regulated (denoted as green) and 285 genes were significantly down-regulated (denoted as red), in the *fixLJ* deletion mutant relative to AU0158. A sampling of selected genes meeting these criteria is shown in Table 1, and the full list of these genes is in S1 Table. Genes appearing in Table 1 were chosen for their magnitude of change and/or potential role in virulence based on roles in related bacteria. We confirmed differential expression of a subset of these genes using qRT-PCR (S4 Fig). In those experiments, we also confirmed that the differential expression could be complemented in the *fixLJ* deletion mutant carrying *fixLJ* compared to the empty vector (S4 Fig). We found that *fixK* was significantly down-regulated in *fixLJ* deletion mutant compared to the parental AU0158 by qRT-PCR (S4 Fig). We found that *fliC* (BDAG_00084, AK34_2913) expression was significantly down-regulated in the *fixLJ* deletion mutant (S4B Fig), which correlated with the reduction in motility seen in Fig 5. We also found that the master regulator of flagella assembly, *flhD* (AK34_2903), was expressed significantly lower in the *fixLJ* deletion mutant compared to the wild-type strain AU0158. Components of a putative type IVa pilus (AK34_2364- AK34_2368) were down-regulated in the *fixLJ* deletion mutant (Table 1). In contrast, multiple subunits of a putative type IVb pilus (AK34_1641- AK34_1653) were actually up-regulated in the *fixLJ* deletion mutant relative to the wild-type strain AU0158, and this was confirmed for the *flp* gene in the qRT-PCR experiments (S4C Fig). A putative type III secretion system (AK34_3647- AK34_3658) was also up-regulated in *fixLJ* deletion mutant (Table 1). Notably, several transcription regulators were significantly up- or down-regulated indicating that the *fix* regulon is complex.

**Table 1:** Selected genes of interest that were significantly differentially regulated in the *fixLJ* deletion mutant relative to the wild-type strain AU0158 measured by RNA-seq^a^.

Category/Gene name (genome designation)	Description	Fold change in *fixLJ* deletion mutant
Motility		
* flhD* (AK34_2903)	Flagellar transcriptional activator	-2.9
* cheY* (AK34_2899)	Chemotaxis regulator	-2.1
* *AK34_269	methyl-accepting chemotaxis (MCP) signaling domain protein	-2.1
Type IVa pilus		
* pilE* (AK34_2364)	Type IV pilus biogenesis protein	-2.7
* *AK34_2365	FIG00453045: hypothetical protein	-4.4
* pilW* (AK34_23660	Type IV fimbrial biogenesis protein	-9.7
* fimT* (AK34_2368)	Tfp pilus assembly protein FimT	-3.1
Type IVb pilus		
* *AK34_1641	Response regulator containing CheY-like receiver	3.1
* tadD* (AK34_1644)	Flp pilus assembly protein	2.3
* tadC* (AK34_1645)	Type II/IV secretion system protein	2.4
* tadA* (AK34_1647)	Type II/IV secretion system ATP hydrolase	2.7
* * AK34_1649	Type II/IV secretion system secretin RcpA/CpaC	2.4
* *AK34_1652	Probable prepilin peptidase transmembrane protein	2.1
* flp* (AK34_1653)	Flp pilus assembly protein, pilin Flp	2.1
Type III Secretion System		
* *AK34_3647	Type III secretion bridge between inner and outer membrane lipoprotein	10.1
* *AK34_3648	Type III secretion cytoplasmic protein	10.5
* *AK34_3641	Type III secretion inner membrane channel protein	9.4
* *AK34_3649	Type III secretion cytoplasmic protein	8.6
* *AK34_3650	Type III secretion cytoplasmic ATP synthase	7.9
* *AK34_3657	Type III secretion inner membrane protein	8.0
* *AK34_3658	Type III secretion inner membrane protein	6.8
Regulators		
* *AK34_1304	RNA polymerase sigma factor RpoS	5.0
* *AK34_3568	Transcriptional regulator, LysR family	3.4
* *AK34_1422	Transcriptional regulator, LysR family	3.1
* *AK34_3852	Transcriptional regulator, MarR family	-6.1
* *AK34_5402	Transcriptional regulator, LysR family	-5.4
* *AK34_2833	Transcriptional regulator, AsnC family	-3.6
* *AK34_1626	Transcriptional regulator/sugar kinase	-9.5
* *AK34_4350	Two component system histidine kinase	-10.5
Other		
* rbsK* (AK34_5286)	Ribokinase	-9.1
* * AK34_4362	Putative cytochrome P450 hydroxylase	-44.7
* * AK34_1628	ABC-type sugar transport system, permease protein	-9.4
* * AK34_1627	ABC-type sugar transport system, ATP-binding protein	-9.9
* * AK34_5433	Outer membrane protein (porin)	-3.6
* * AK34_3420	Major facilitator family transporter	-3.6
* * AK34_1355	Outer membrane protein W precursor	11.4
* * AK34_3656	Peptidoglycan hydrolase VirB1	11.6
* * AK34_2361	Probable transmembrane protein	3.1
* hfq* (AK34_1317)	RNA-binding protein	2.8
* * AK34_4984	FOG: GGDEF domain	2.4
* * AK34_1354	Dioxygenases related to 2-nitropropane dioxygenase	5.8
* * AK34_3566	Enoyl-CoA hydratase	13.6
* * AK34_3563	Acetyl-CoA synthetase	13.9
* * AK34_3562	Butyryl-CoA dehydrogenase	15.8
* * AK34_3722	4-carboxymuconolactone decarboxylase	11.9
* * AK34_3721	Phenylacetic acid degradation protein PaaY	13.0
* * AK34_4113	Outer membrane protein (porin)	-4.7

^a^ Significance is defined as greater than 2-fold change with *q* <0.05.

### Presence of *B*. *dolosa* flagella does not affect virulence

**Fig 7:**
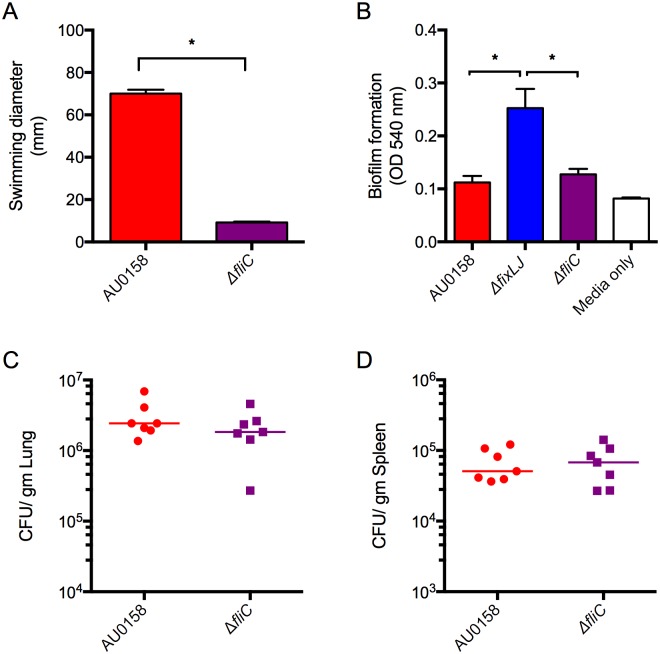
*B*. *dolosa* virulence is independent of the presence of flagella. (A) *B*. *dolosa* strain AU0158 and its *fliC* deletion mutant were plated on low-density (0.3%) LB agar and swimming distance was measured after incubation for 48 hours. *P<0.05 by 1-way NOVA with Tukey’s multiple comparison test. Bars represent mean measurements of 3–4 replicates and error bars represent one standard deviation (representative of three independent experiments). (B) Overnight cultures of *B*. *dolosa* strain AU0158, the *fixLJ* deletion mutant, or the *fliC* deletion mutant were diluted 1:100 in TSB with 1% glucose, incubated for 48 hours, and then assessed for biofilm formation using crystal violet. Bars represent mean measurements of 6 replicates and error bars represent one standard deviation of the data (representative of three independent experiments). (C-D) C57BL/6 mice were intranasally challenged with 4.8 x10^8^ CFU/mouse of strain AU0158 or its *fliC* deletion mutant. Bacterial loads were measured 7 days after infection in the lungs (C) and spleen (D). Data are derived from one experiment done with 7–8 mice per group. Each point represents one mouse and bars represent medians.

Since other groups have found that flagella and motility are associated with virulence in *B*. *cenocepacia* [28, 29] and we found decreased motility (Fig 5) and decreased expression of flagella-associated genes (Table 1 and S4 Fig) in the *fixLJ* deletion mutant, we tested the hypothesis that the decreased expression of the flagella in the *fixLJ* deletion mutant is a major mechanism for the reduction of *in vivo* persistence seen after lung infection with this mutant (Fig 3). To do this, we generated a *fliC* deletion mutant in *B*. *dolosa* strain AU0158 background. As expected this mutant was completely non-motile when plated on low-density LB agar plates (Fig 7A). We also saw no difference in the ability of the *fliC* deletion mutant to form biofilm on PVC plates after 48 hours compared to strain AU0158 (Fig 7B). We next infected C57BL/6 mice with ~5x10^8^ CFU/mouse of strain AU0158 or its *fliC* deletion mutant via the intranasal route and examined bacterial loads in the lungs and spleen 7 days after infection. There was no statistically significant difference in levels of viable bacteria in the lungs (Fig 7C) or the spleen (Fig 7D).

### *B*. *dolosa fixLJ* deletion mutants are less invasive

**Fig 8:**
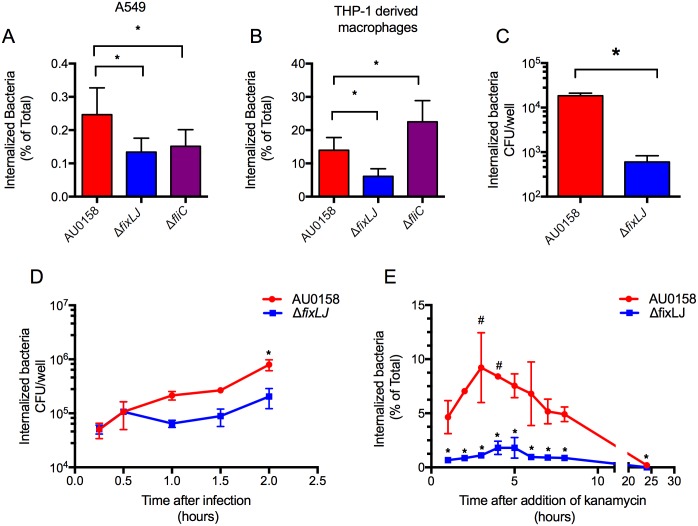
The *B*. *dolosa fixLJ* deletion mutant is less invasive of epithelial and macrophage-like cells. A549 cells (A) or THP-1 cells treated with 200 nM PMA for 3 days (B) in 24-well plates were infected with ~2x10^6^ CFU/well (MOI of ~10:1) of strain AU0158, its *fixLJ* deletion mutant, or its *fliC* deletion mutant for 2 hours, after which the percent of internalized bacterial relative to the total bacterial growth was determined by killing extracellular bacteria with kanamycin (1 mg/mL). Means from 2–3 separate experiments with three replicates per experiment are plotted with error bars representing one standard deviation. *P<0.05 by 1-way ANOVA with Tukey’s multiple comparison test. (C) THP-1-derived macrophages were infected with ~2x10^6^ CFU/well of *B*. *dolosa* for 2 hours, after which the extracellular bacteria were killed by treatment with kanamycin (1 mg/mL) and number of intracellular bacteria were determined after a 24-hour incubation. *P<0.05 by t test. (D) THP-1-derived macrophages were infected with ~2x10^6^ CFU/well of *B*. *dolosa* for varying amounts of time (15 min-2 hours) after which the number of internalized bacteria was determined by killing extracellular bacteria with kanamycin (1 mg/mL). *P<0.05 by t test compared to the *fixLJ* deletion mutant at that time point. (E) THP-1-derived macrophages were infected with ~2x10^6^ CFU/well of *B*. *dolosa* for 2 hours, after which the extracellular bacteria were treated with kanamycin (1 mg/mL) for varying amounts of time, after which the percent of internalized bacterial relative to the total bacterial growth within the initial 2-hour infection was determined. *P<0.05 by t test compared to strain AU0158 at that time point. # *P*<0.05 by 1-way ANOVA with Tukey’s multiple comparison test compared to hour-1 measurement. (C-E) Means from representative experiment repeated twice. Separate experiments with three replicates per experiment are plotted with error bars representing one standard deviation.

Since we observed multiple pili systems and flagella associated genes to be differentially expressed in the *fixLJ* deletion mutant in our RNA-seq experiments (Table 1, S1 Fig, S1 Table) we next determined the ability of the *fixLJ* deletion mutant to invade and persist within A549 lung epithelial cells and THP-1-derived macrophages. Multiple pili systems and flagellum have been described to be involved in invasion in BCC and other ands Gram-negative bacteria [29–32]. We infected A549 cells or THP-1-derived macrophages with *B*. *dolosa* (M.O.I. of ~10:1) for two hours, after which extracellular bacteria were killed by the addition of kanamycin. There was ~2-fold reduction in the amount of *fixLJ* deletion mutant bacteria within A549 lung epithelia cells compared to the parental strain, AU0158 (Fig 8A). We also found that the *fixLJ* deletion mutant has ~4 fold reduction compared to parental AU0158 in the ability to survive within THP-1 cells that had been previously treated with PMA to induce differentiation into macrophage-like cells (Fig 8B). Similar to the *fixLJ* deletion mutant, we found a ~2-fold reduction in invasiveness of the *fliC* deletion mutant compared to strain AU0158 in A549 lung epithelial cells (Fig 8A). In contrast, when THP-1 derived macrophages were infected with *fliC* deletion mutant, we saw an increase in the number of intracellular bacteria relative to strain AU0158 (Fig 8B). To understand the mechanism of decreased bacterial loads in THP-1 derived macrophages infected with the *fixLJ* deletion mutant, we varied the amount of time the bacteria had to invade before kanamycin treatment or the time of kanamycin treatment. When infected THP-1 derived macrophages were treated with kanamycin for 24 hours to kill extracellular bacteria, there was a 30-fold reduction in the number bacteria inside *fixLJ* deletion mutant-infected cells compared to AU0158-infected cells (Fig 8C). To determine if the *fixLJ* deletion mutant is less able to become internalized than strain AU0158, we varied the length of infection time before the addition of kanamycin. We found that both the *fixLJ* deletion mutant and the parental AU0158 could equally become internalized at the early time points (15 & 30 min, Fig 8D) and there was no significant difference until 2 hours (Fig 8D). When we did the complementary experiment to measure the ability of the bacteria to replicate intracellularly by varying the amount of time of kanamycin exposure after a 2 hour infection period, we found there was a significant ~2-fold increase in the number of intracellular bacteria at 3 and 4 hours post infection in THP-1 derived macrophages infected with strain AU0158 relative to 1 hour post infect that was not seen in macrophages infected with the *fixLJ* deletion mutant (Fig 8E). Across all time points, there were significantly greater numbers of bacteria in strain AU0158-infected cells compared to cells infected with the *fixLJ* deletion mutant (Fig 8E). To address the possibility of the *B*. *dolosa* constructs causing differing amounts of cytotoxicity, we measured the cytotoxic effects of the live bacteria on A549 and THP-1 derived macrophages. We saw minimal amounts of LDH release above unstimulated cells and found no statistically significant difference in cells infected with strain AU0158 versus the *fixLJ* deletion mutant (*ΔfixLJ*), *ΔfixLJ* + *fixLJ*, *ΔfixLJ* + empty vector (EV), or strain AU0158 vs. the *fliC* deletion mutant in LDH release after 6 hours of exposure (S5 Fig).

## Discussion

A recent study using whole-genome sequencing of multiple strains from an outbreak of *B*. *dolosa* in CF patients at Boston Children’s Hospital discovered that the *fixLJ* genes show evidence of strong positive selection. These findings were recently corroborated to another BCC species, *B*. *multivorans* [20]. In the current study, we report the functional significance of the *fixLJ* genes in *B*. *dolosa*, finding not only that the pathway is induced by low oxygen, but also that it regulates a large number of genes and is critical for pathogenicity *in vivo* and intracellular invasion *in vitro*.

Our findings that the *B*. *dolosa fix* pathway is activated by low oxygen fit well with prior reports that the *B*. *cenocepacia* homolog of *fixK* (BCAM0049) was up-regulated during growth in a low-oxygen environment [18, 19]. FixL homologs in other pathogenic Gram negative bacteria include one in *Brucella*, which was found to be necessary for intracellular survival [33]. *Pseudomonas aeruginosa* has a FixL homolog called BfiS (biofilm initiation sensor) along with a FixJ homolog, BfiR, which, as their names suggest, have been shown to be critical for the irreversible attachment phase of biofilm development [34, 35]. The environmental signal that activates BfiS has not yet been elucidated. One recent study using Tn-seq found that BfiS and BfiR were critical for virulence in a murine model of GI tract colonization as fitness was 24- or 60-fold reduced compared to *in vitro* grown bacteria, respectively, and BfiS and BfiR transposon mutants were unable to disseminate after these colonized mice were made neutropenic [36].

We found that the *fixLJ* deletion mutant was less able to persist within the murine model (Fig 2). At 1 day after infection, we saw lower numbers of bacteria within the lungs but similar levels of in the spleens of mice infected with the *fixLJ* deletion mutant, demonstrating that there was no defect in the ability of the *fixLJ* deletion mutant to disseminate. Dissemination from the lungs to the spleen could occur via an intracellular or an extracellular mechanism. In Fig 8, we show that the *fixLJ* deletion mutant was less able to survive within THP-1 derived macrophages, which suggests that *fixLJ* deletion mutant will be less able to disseminate via an intracellular mechanism. These findings suggest that *B*. *dolosa* dissemination occurs via an extracellular mechanism in this model. Lung histology showed evidence of significant inflammation, consistent with the bacteria causing an infection opposed to colonization (S2 Fig). There was no difference in lung pathology induced by strain AU0158 versus the *fixLJ* deletion mutant, but this is likely related to the relatively high bacterial load seen in both infection groups.

Interestingly the *B*. *dolosa fixLJ* deletion mutant made significantly more biofilm than the parental strain, which is the opposite of what was seen when the *P*. *aeruginosa bfiSR* pathway was inactivated [34]. Confocal microscopy images were consistent with the crystal violet staining, where the *fixLJ* deletion mutant produced more biofilm likely due to the increased number of bacteria attached to the glass (Fig 4). These results are interesting because other groups have shown that non-motile *P*. *aeruginosa* and BCC are less likely to form biofilms [37, 38], although non-motile BCC seem to be able to produce similar amounts of biofilm compared motile wild-type strains if given enough time [38]. The clinical relevance of BCC biofilm production is unclear. Indeed, one study found no correlation between the ability of BCC isolates to form biofilm and clinical outcomes [39]. In another study that examined *P*. *aeruginosa* and/or BCC infected CF lung tissue removed after transplant using species-specific antibodies to stain the lung tissue, BCC bacteria were rarely found in biofilm-like structures while *P*. *aeruginosa* were often found in such structures [40]. These reports along with our findings suggest that biofilm production may not be beneficial for BCC infection.

Using RNA-seq, we found that ~11% of the *B*. *dolosa* genome was differentially expressed within the *fixLJ* deletion mutant, demonstrating the size of the FixLJ regulon. Multiple components of the flagella assembly pathway were found to be down-regulated in the *fixLJ* deletion mutant. To evaluate the reduction in flagella expression as the mechanism of reduced persistence *in vivo* seen in the *fixLJ* deletion mutant, we also tested a *fliC* deletion mutant. We found that there was no reduction in the ability of the *fliC* deletion mutant to persist in the murine lung or spleen, demonstrating that flagella are not required for *B*. *dolosa* virulence in our model. This was a surprising result as other groups have found that BCC lacking flagella (or otherwise non-motile) are less invasive, less able to form biofilms, and less lethal in murine models [28, 29, 41]. This finding suggests that the virulence factors and mechanism of infection of *B*. *dolosa* are in some respects different from other members of the BCC.

The ability of BCC to invade and/or survive within epithelial cells and macrophages is thought to represent an important aspect of BCC pathogenesis [42–44]. We found that the *B*. *dolosa fixLJ* deletion mutant was less invasive towards A549 epithelial cells and THP-1 derived macrophages (Fig 8). It is possible that the *fixLJ* deletion mutant is either less able to invade the cell or less able to survive within the cell, or both. We found that the *fixLJ* deletion mutant could equally become internalized as the parental AU0158 at early time points while the *fixLJ* deletion mutant was unable to replicate intracellularly. The decreased numbers of the *fixLJ* deletion mutant seen at the late time points in Fig 8D is likely due to decreased ability of the *fixLJ* deletion mutant to survive and/or replicate intracellularly. These findings suggest that lower intracellular survival seen in the *fixLJ* deletion mutant is responsible for the majority of the decrease in invasiveness seen in Fig 8A and 8B. We hypothesize that the *fixLJ* deletion mutant is unable regulate appropriate gene expression to survive within the intracellular environment since it is unable to detect the lower oxygen concentration found there.

Compared to strain AU0158, the *fliC* deletion mutant was less able to invade/survive within A549 cells, but it was better able to survive within THP-1 derived macrophages. Both cell types were centrifuged after addition of bacteria, eliminating the possibility of the *fliC* deletion mutant being unable to swim to the human cell. Mohr and co-workers also reported that *B*. *cepacia* flagella deletion mutants were less able to invade A549 cells [29]. Our finding that the *B*. *dolosa fliC* deletion mutant had an increased number of intracellular bacteria in THP-1 derived macrophages likely relates to the absence of TLR-5 mediated activation of macrophages by flagella in the *fliC* deletion mutant. Our data overall suggest that the ability of *B*. *dolosa* to survive within THP-1 derived macrophages is a better surrogate than survival within A549 epithelial cells for *in vivo* fitness since results with the THP-1 derived macrophages matched well with the murine model (*fixLJ* deletion mutant attenuated but *fliC* deletion mutant not attenuated).

Our RNA-seq data suggest other pathways that might explain the phenotypes seen in the *fixLJ* mutant. We found genes encoding a putative type IVa pilus that is downregulated in the *fixLJ* deletion mutant. A similar type IVa pilus in *Burkholderia pseudomallei* has been described to bind to human epithelial cells and be required for virulence in nematode and murine models [30]. Other studies have found the homolog of the pilin (*pilE*) that was down-regulated in the *fixLJ* deletion mutant to be critical for attachment of pathogenic *Neisseria* species to human cells [31, 32]. We also identified genes encoding a putative type IVb pilus that is upregulated in the *fixLJ* deletion mutant. Type IVb pili have been described to be involved in tight adhesion to substrates and biofilm formation [45], so this pathway might explain the high biofilm production of the *fixLJ* deletion mutant. Consistent with this hypothesis, we found that the *fixLJ* deletion mutant biofilm was susceptible to proteinase K treatment, suggesting dependency on proteins such as the flp pilus for biofilm formation. Further supporting this hypothesis, we found that the *fixLJ* deletion mutant was able to adhere to the entirety of the glass surface, which is perhaps due to the increased expression of the flp pilus in the *fixLJ* deletion mutant compared to strain AU0158, which primarily formed biofilms with dome-like structures (Fig 4). Interestingly, homologs of the up-regulated component of the type IVb pilus (*flp* subunit) were found to be down-regulated *in vivo* in a chronic *B*. *cenocepacia* infection model in rats [46], suggesting that this pilus is detrimental to *in vivo* fitness during long-term infections. We also saw that the RNA-binding protein Hfq was upregulated in the *fixLJ* deletion mutant, suggesting a role for sRNA in modulation of the *fix* regulon. We also found a large number of regulatory proteins, suggesting the *fixLJ* regulon is complex and composed of elements that are both directly and indirectly regulated by *fixLJ*.

We also used the RNA-seq data to examine pathways that have been identified in other BCC to mediate the phenotypes we observed. We found 2 sigma factors, *rpoN* (AK34_313) and *rpoE* (AK34_2044), which were 1.9 and 2.3, respectively upregulated in the *fixLJ* deletion mutant relative to AU0158. Both of these sigma factors were found to be critical for *B*. *cenocepacia* survival within macrophages, and *rpoN* was also critical for biofilm formation [41, 47]. In Table 1 we identify a putative T3SS that was upregulated in the *fixLJ* deletion mutant relative to AU0158. Tomich *et al*. found that *B*. *cenocepacia* T3SS mutants are less fit a murine model [48]. In both cases we would have expected the sigma factors and T3SS to be downregulated in the *fixLJ* deletion mutant if they were responsible for the phenotypes observed. The fact that these previously published findings are not consistent with our findings demonstrates that these phenotypes are complex, with multiple factors involved, and warrant further investigation. BCC produce an exopolysaccharide, cepacian, that is required for mature biofilm formation [39]. We saw no differential expression in any of the genes involved in the production of cepacian [49] suggesting that other mechanisms such as the flp pilus are responsible for the increased biofilm production.

Whole-genome sequencing of isolates from an outbreak of *B*. *dolosa* led to the identification of the *fixLJ* pathway being under strong positive selection during chronic infection [15, 50]. In this study, we suggest that the *B*. *dolosa* FixLJ system is an oxygen-sensing mechanism that regulates biofilm formation, motility, intracellular invasion, and virulence. It appears that *fixLJ* is required for *in vivo* persistence by limiting biofilm formation and allowing for survival within macrophages, which is known to be a low-oxygen environment. Future studies will better characterize the FixLJ pathway and identify individual components that are required for *in vivo* persistence.

## Materials and Methods

### Ethics statement

All animal protocols and procedures were approved by the Boston Children’s Hospital Institutional Animal Care and Use Committee (assurance number A3303-01). The specific protocol number is 15-02-2889. All animal protocols are complement with NIH Office of Laboratory Animal Welfare, Guide for the Care and Use of Laboratory Animals, The US Animal Welfare Act, and PHS Policy on Humane Care and Use of Laboratory Animals.

### Bacterial strains, plasmids, cell lines, and growth conditions

All strains used and generated in this study are listed in Table 2. *B*. *dolosa* strain AU0158 was obtained from John LiPuma (University of Michigan) and is an early isolate from the index patient from the *B*. *dolosa* outbreak (about 3 years into the outbreak). BCC and *E*. *coli* were grown on LB plates or in LB medium and supplemented with following additives: ampicillin (100 μg/mL), kanamycin (50 μg/mL for *E*. *coli*, 1 mg/mL for BCC), trimethoprim (100 μg/mL for *E*. *coli*, 1 mg/mL for BCC), gentamicin (15 or 50 μg/mL), chloramphenicol (20 μg/mL), or diaminopimelic acid (200 μg/mL). Plasmids that were used in this study are listed in Table 3. Human lung epithelial cells A459 and human monocyte line THP-1 were obtained from ATCC. Both cell lines were grown at 37°C with 5% CO_2_. A459 cells were grown in RPMI 1640 with L-glutamine and 10% heat-inactivated fetal calf serum (FCS, Gibco). Penicillin and streptomycin were added for routine culture but were removed the day before and during experiments. THP-1 cells were cultured in RPMI-1640 medium containing 2 mM L-glutamine, 10 mM HEPES, 1 mM sodium pyruvate, 4500 mg/L glucose, and 1500 mg/L sodium bicarbonate, supplemented with 10% heat-inactivated FCS and 0.05 mM 2-mercaptoethanol. Human THP-1 monocytes were differentiated into macrophages by seeding 1 mL into 24 well plates at 8x10^5^ cells/mL with 200 nM phorbol 12-myristate 13-acetate (PMA) and incubated for 72 hours then washed two times with media lacking PMA. Low oxygen environments were generated by the CampyGen Gas Generating System (Thermo-Fisher), and the low-oxygen concentration (<5%) is based on the manufacture specifications.

**Table 2:** Strains used in this study.

	Notes	Source
*E*.*coli*		
NEB 5-alpha Competent *E*. *coli*	DH5α derivative cloning strain	NEB
* *RHO3	Mobilizer strain. Km^s^; SM10(λ*pir*) Δ*asd*::*FRT* Δ*aphA*::*FRT*	[51]
BCC		
* B*. *dolosa* AU0158	Clinical isolate	John LiPuma
* B*. *cenocepacia* J2315	Clinical isolate	Joanna Goldberg
* B*. *multivorans* ATCC 17616	Clinical isolate	ATCC
* B*. *dolosa* Δ*fixLJ*	*B*. *dolosa* AU0158 *fixLJ* deleted	This study
* *Δ*fixLJ* + *fixLJ*	*B*. *dolosa* Δ*fixLJ* + pfixLJ integrated at *att*Tn7 site downstream of BDAG_4221	This study
* *Δ*fixLJ* + EV	*B*. *dolosa* Δ*fixLJ* + empty pUC18T-mini-Tn*7*T-Tp integrated at *att*Tn7 site downstream of BDAG_4221	This study
* *Δ*fixLJ* + *fixLJ-*rha	*B*. *dolosa* Δ*fixLJ* + pfixLJrha integrated at *att*Tn7 site downstream of BDAG_4221	This study
* *Δ*fixLJ* + EV-rha	*B*. *dolosa* Δ*fixLJ* + empty pTJrha integrated at *att*Tn7 site downstream of BDAG_4221	This study
* B*. *dolosa /* pfixK-reporter	*B*. *dolosa* carrying pfixK-reporter plasmid	This study
* B*. *dolosa /* pSmfixK-reporter	*B*. *dolosa* carrying pSmfixK-reporter -reporter plasmid	This study
* B*. *cenocepacia/* pfixK-reporter	*B*. *cenocepacia* J2315 carrying pfixK-reporter plasmid	This study
* B*. *multivorans* / pfixK-reporter	*B*. *multivorans* ATCC 17616 carrying pfixK-reporter plasmid	This study
* *Δ*fixLJ* + *fixLJ* / pfixK-reporter	*B*. *dolosa* Δ*fixLJ* complemented with *fixLJ* carrying pfixk-reporterKm	This study
* *Δ*fixLJ* + EV/ pfixK-reporter	*B*. *dolosa* Δ*fixLJ* complemented with empty vector carrying pfixk-reporterKm	This study
*B*. *dolosa* Δ*fliC*	*B*. *dolosa* AU0158 *fliC* deleted	This study

**Table 3:** Plasmids used in this study.

	Notes	Source
pEXkm5	Km^R^, *sacB*, *gusA*	[51]
pFIXKO	pEXkm5 carrying flanking regions of *fixLJ*	This study
pRK2013	Km^R^ conjugation helper	[52]
pSCrhaB2	Tp^R^, *ori*_pBBR1_*rhaR*, *rhaS*	[26]
pUC18T-mini-Tn*7*T-Tp	Amp^R^, Tp^R^ on mini-Tn*7*T; mobilizable	[52]
pfixLJ	pUC18T-mini-Tn*7*T-Tp carrying *fixLJ* with 670 upstream flanking	This study
pTJ-1	Amp^R^, Tp^R^, pUC18T-mini-Tn*7*T-Tp-*araC*-PBAD-MCS;	[53]
pTJrha	pTJ-1 with rhamnose operator in place of arabinose operator	This study
pFixLJrha	pTJrha with *fixLJ* under the control of rhamnose operator	This study
pTNS3	Amp^R^, helper plasmid for mini-Tn7 integration into *att*Tn7 site	[54]
*S*. *meliloti* P*fixK-lacZ* reporter plasmid	Cm^R^, *S*. *meliloti fixK-*controlled expression of *lacZ*	[21]
pSmfixK-reporter	pSCrhaB2 carrying *S*. *meliloti fixK-lacZ* fusion	This study
pfixK-reporter	pSCrhaB2 carrying *B*. *dolosa fixK-lacZ* fusion	This study
pfixk-reporterKm	KmR pfixK-reporter with Km^R^ in place of Tp^R^	This study
pEXKm5-fliCdel	pEXKm5 carrying flanking regions of *fliC*	This study
pEXKm5Tet-fliCdel	pEXKm5-fliCdel with *tet*^*R*^	This study

### Genetic manipulations and strain construction

An in-frame, clean deletion of *fixLJ* in *B*. *dolosa* AU0158 was generated using the suicide plasmid pEXKm5 [51]. Briefly, 1.4 kbp and 1.1 kbp flanking upstream and downstream, respectively, were PCR amplified to introduce *Sma*I sites on the 5’ and 3’ ends of the upstream and downstream fragments, respectively, and 16 bp overlap between the 3’ end of the upstream fragment and the 5’ end of the downstream fragment. The two fragments were joined using the NEBuilder HiFi DNA Assembly Master Mix (NEB) per manufacturer’s protocol and sub-cloned into pGEM-T (Promega). The fragment was digested with *Sma*I and cloned into pEXKm5 at the *Sma*I site and was named pFIXKO. pFIXKO was Sanger sequenced and transformed into RHO3 *E*. *coli* and then conjugated into *B*. *dolosa* AU0158 along with the conjugation helper plasmid pRK2013 [54, 55]. Conjugants were selected for on LB with kanamycin at 1 mg/mL. Insertion of the plasmid was confirmed by PCR. To resolve merodiploidy, conjugants were counterselected against by plating on LB with 15% w/v sucrose and incubated for 2 days at 30°C. Resolution and confirmation of *fixLJ* deletion was confirmed by PCR and Sanger sequencing.

To complement the *fixLJ* deletion mutant, we generated stable chromosomally integrated constructs using the mini-Tn7 system, which integrates into an *att*Tn7 site [52, 55]. *B*. *dolosa* strain AU0158 *fixLJ* along with 670 bp upstream was amplified using PCR with primers that generated *Nsi*L and *Kpn*I sites at the 5’ and 3’ ends respectively. This fragment was cloned into pUC18T-mini-Tn*7*T-Tp at the *Nsi*L and *Kpn*I sites, generating pfixLJ. A rhamnose-inducible FixLJ expression construct was generated by replacing the arabinose operator of pTJ-1 with rhamnose operator of pSCrhaB2 using NEBuilder HiFi DNA Assembly Master Mix (NEB) per manufacturer’s protocol and primers generated using NEBuilder Assembly tool, generating pTJrha. *B*. *dolosa* AU0158 *fixLJ* was amplified by PCR with primers that generated *Nco*I and *Hind*III sites at the 5’ and 3’ ends respectively. This fragment was cloned into pTJrha at the *Nco*I and *Hind*III sites generating the plasmid pFixLJrha. All insertions were verified by PCR and Sanger sequencing. The *fixLJ* complementation vectors and the corresponding empty vector controls were conjugated into the *fixLJ* deletion mutant with pRK2013 and pTNS3 using published procedures [54, 55]. Conjugants were selected for by plating on LB agar containing trimethoprim (1 mg/mL) and gentamicin (50 μg/mL). Insertions into the *att*Tn7 site downstream of BDAG_4221 was confirmed by PCR.

To generate the *fixK-lacZ* reporter plasmid the first 23 bp of *fixK* and the immediate 243 bp upstream of the start codon were PCR amplified to generate a *Hind*III and *Kpn*I sites at the 5’ and 3’ ends respectively. The fragment was subcloned into the *Hind*III and *Kpn*I sites of *S*. *meliloti* P*fixK-lacZ* reporter plasmid [21] generating an in-frame fusion of P*fixK* with *lacZ*. The *S*. *meliloti* and *B*. *dolosa* P*fixK-lacZ* fusion was removed by digestion with *Hind*III and *Bam*HI, and cloned into pSCrhaB2 at the *Hind*III and *Bam*HI sites which resulted in pSmfixK-reporter and pfixK-reporter, respectively. The insertion was verified by PCR and occurred in the opposite orientation of the rhamnose operator, which is in pSCrhaB2. For use in some experiments the trimethoprim resistance gene of pfixK-reporter in the pSCrhaB2 backbone was replaced with the kanamycin resistance gene of pEXKm5 using NEBuilder HiFi DNA Assembly Master Mix (NEB) per manufacturer’s protocol and primers generated using NEBuilder Assembly tool generating pfixk-reporterKm. The pfixK-reporter and pSmfixk-reporter were conjugated into *B*. *dolosa* AU0158, *B*. *cenocepacia* J2315, and *B*. *multivorans* ATCC 17616 in triparental matings with pRK2013 [54, 55]. Conjugants were selected for by plating on LB agar containing trimethoprim (1 mg/mL) and gentamicin (50 μg/mL). Conjugants were maintained in LB containing trimethoprim (1 mg/mL).

An in-frame, clean deletion mutation in the *B*. *dolosa fliC* gene was generated using similar methods. Briefly, 700 bp upstream and 700 bp downstream of BDAG_00084 was amplified by PCR. These templates were used in a second PCR as templates to generate fused amplicons. These amplicons were restricted and ligated into in the *Xma*I and *Eco*RI sites of pEXKm5, generating pEXKm5-fliCdel. For use in *B*. *dolosa* we cloned in the *tet* gene from miniCTX plasmid ligated into the *Spe*I sites of pEXKm5-fliCdel. Sm10λ were transformed with pEXKm5Tet-fliCdel and then transferred into *B*. *dolosa* by conjugation, and complemented isolates were counterselected on LB agar supplemented with kanamycin (50 μg/ml) + tetracycline (75 μg/ml) + ampicillin (100 μg/ml). Insertion of the plasmid into the chromosome was verified by PCR. Counter-selection, to resolve merodiploidy was performed by plating the transconjugants on LB medium + 10% sucrose. Resolution and confirmation of *fliC* deletion was confirmed by PCR and Sanger sequencing.

### Reporter assay

*Burkholderia* carrying a *fixK-lacZ* report plasmid were grown overnight in LB with kanamycin or trimethoprim (1 mg/mL). Cultures were subcultured in LB in ambient air or LB that had been degassed in CampyGen Gas Generating System (Thermo-Fisher). Cultures were grown in ambient oxygen with shaking (200 rpm) at 37°C or within CampyGen Gas Generating System at 37°C for 4–6 hours. The level of *fix-*driven LacZ activity was measured by determining Miller Units following published procedures [21].

### Murine model of pneumonia

Female C57BL/6 mice 6–8 weeks of age were obtained from Taconic Biosciences. Mice were maintained at the animal facilities at Boston Children’s Hospital. Mice were anesthetized with ketamine (100 mg/kg) and xylazine (13.3 mg/kg) given intraperitoneally. While the mice were held in dorsal recumbency, 10 μL of inoculum was instilled in each nostril (20 μL total). The inoculum consisted of log-grown *B*. *dolosa* washed in PBS and diluted to a concentration of ~2x10^10^ CFU/mL (4x10^8^ CFU/mouse). Mice were euthanized 1 or 7 days after infection by CO_2_ overdose, when lungs and spleen were aseptically removed. Lungs and spleens were weighed and placed into 1 mL protease peptone, homogenized, and then serially diluted and plated on Oxidation/Fermentation-Polymyxin-Bacitracin-Lactose (OFPBL) plates. At 7 days after infection, mice infected in parallel were euthanized by CO_2_ overdose; 1 mL 1% paraformaldehyde (Boston BioProducts) was instilled into the lungs for 5 min. Lungs were removed and fixed in 1% paraformaldehyde for 2 hours. Lungs were stored in 70% ethanol until paraffin embedded and 5 micron sections were made and hematoxylin and eosin stained. A rodent pathologist scored the slides for degree of lung inflammation.

### Biofilm formation

The ability to form biofilm on PVC plates was determined using published methods [56]. Briefly overnight cultures were diluted in TSB with 1% glucose and pipetted into wells of a 96-well PVC plate. Plates were incubated 48 hours at 37°C, when unattached bacteria were washed with DI water. Biofilms were stained with 0.5% crystal violet and excess stain was washed away, and stain was solubilized with 33% acetic acid. The solution was transferred to a flat bottom plate, and then staining (biofilm amount) was quantified by measuring O.D._540_. To measure biofilm dispersal, 48 hour biofilms were washed with DI water and treated with 120 u/mL DNase I, 3.18 mAu/mL proteinase K, or 50 μg/mL dispersin B for 24 hours at 37°C. Biofilm was then measured as described above.

### Confocal microscopy

Overnight cultures of strain AU0158 or its *fixLJ* deletion mutant were diluted 1:100 in TSB with 1% glucose and 200 μL was pipetted into 4 wells of an 8-well glass chamber slide (Lab-Tek) and incubated for 48 hours at 37°C. Non-adherent bacteria were removed by two washes with PBS. Bacteria were stained with Live/Dead BacLight Bacterial Viability Kit (Molecular Probes) using STYO 9 and propidium iodide per the manufacturer’s protocol. Stained slides were washed with PBS twice and fixed with 4% paraformaldehyde for 15 minutes at room temperature and then visualized Zeiss Axiovert Spinning Disk Confocal Microscope. Mosaic images consisting of 16 fields of view and 1 μm Z-stack images were acquired analyzed using Slidebook (3i, Intelligent Imaging Innovations Inc.).

### Motility assay

The ability *B*. *dolosa* to swim was measured in low density LB agar using published methods [57]. Briefly, 10 μL of overnight *B*. *dolosa* culture was plated in the center of low density (0.3% agar) LB plate. Plates were incubated agar side down for 48 hours at 37°C when swimming diameter was measured.

### RNA-seq

RNA was isolated from log phase *B*. *dolosa* (two biological replicates for AU0158 and three biological replicates for AU0158 *fixLJ* deletion mutant) using the Ribopure Bacterial RNA Purification Kit (Ambion) per manufacturer’s protocol, contaminant DNA was removed using provided DNase. RNA was cleaned using Trizol Reagent (Fisher Scientific) per manufacturer’s protocol. rRNA was depleted from 10 μg of total RNA using the MICROB*Express* Bacterial mRNA Enrichment Kit (Ambion) and then the RNA was fragmented using NEBNext Magnesium RNA Fragmentation Module (NEB), both per manufacturer’s protocols. The NEBNext Multiplex Small RNA Library Prep Set for Illumina was used to prepare the library with each replicate labeled with a unique barcode enabling multiplexing. Samples were sequenced using single end 50 bp reads using the Illumina HiSeq. Data analysis occurred within in the Galaxy platform (usegalaxy.org) [58]. Reads (9–12 million reads per replicate) were trimmed using the Trimmomatic tool [59] and mapped to the *B*. *dolosa* AU0158 genome (GenBank assembly accession GCA_000959505.1) [60] using BowTie2 using very sensitive local preset settings [61]. Differentially expressed genes were identified using CuffDiff using Benjamini–Hochberg procedure to determine the q value (*p* value corrected for multiple comparisons) [62].

### Bacterial invasion assays

The ability of *B*. *dolosa* to invade and persist within human epithelial cells and macrophages was determined using published protocols [42, 63, 64]. A549 cells were grown to confluence in 24-wells plates. Human THP-1 monocytes were differentiated into macrophages by 72 hour PMA treatment. A549 or THP-1 derived macrophages were infected with log-phase grown *B*. *dolosa* washed in RPMI three times at ~2x10^6^ CFU/well (MOI of ~10:1). Plates were spun at 500 g for 5 minutes to synchronize infection and then incubated 15 minutes -2 hours at 37°C with 5% CO_2_. To determine the total number of bacteria, wells were treated with 100 μL of 10% Triton-X100 lysis buffer (final concentration 1% Triton-X100), serially diluted, and plated to enumerate the number of bacteria. To determine the number of intracellular bacteria, separate infected wells were washed two times with PBS and then incubated with RPMI + 10% heat-inactivated FCS with kanamycin (1 mg/mL) for 1–24 hours. Monolayers were washed three times with PBS and lysed with 1% Triton-X100, serially diluted, and plated to enumerate the number of bacteria.

### qRT-PCR

cDNA was synthesized from 2 μg RNA using the ProtoScript II First Strand cDNA Synthesis Kit (NEB) per manufacture’s protocol. cDNA was cleaned using QIAquick PCR Purification Kit (Qiagen). Genes were amplified using oligos listed in S2 Table and FastStart Essential DNA Green Master Mix (Roche) per manufacturer’s protocol. Expression was determined relative to AU0158 normalized by *gyrB* (AK34_3072) or *rpoD* (AK34_4533) expression using the ΔΔCt method [65]. Both *gyrB* and *rpoD* had similar expression by RNA-seq between AU0158 and the *fixLJ* deletion mutant, and these genes have been used to normalize expression in *B*. *cenocepacia* in other studies [49, 66]

### LDH release assay

A549 cells were grown to confluence in 24 wells plates. Human THP-1 monocytes were differentiated into macrophages by 72 hour PMA treatment. A549 or THP-1 derived macrophages were infected with *B*. *dolosa* washed in RPMI three times at ~1x10^7^ CFU/well (M.O.I. of ~100:1). Plates were spun 500 g for 5 minutes to synchronize infection and incubated 6 hours at 37°C with 5% CO_2_. In other experiments, THP-1 derived macrophages were infected with *B*. *dolosa* washed in RPMI three times at ~1x10^6^ CFU/well (M.O.I. of ~10:1). Plates were spun 500 g for 5 minutes to synchronize infection and incubated 2 hours at 37°C with 5% CO_2_. Plates were washed two times with PBS and then incubated with RPMI + 10% heat-inactivated FCS with kanamycin (1 mg/mL) for 24 hours. All plates were spun 500 x g for 5 minutes and supernatants were removed and froze at -80°C. LDH release was measured within supernatants using CytoTox 96 Non-Radioactive Cytotoxicity Assay (Promega) per manufacture’s protocol. For a positive control untreated wells were treated with 10X lysis buffer (provided with kit) during the last 30 minutes of incubation. Percent cytoxicity was determined relative to maximum LDH release from cells treated with lysis buffer.

## Supporting Information

S1 Fig The *B*. *dolosa fixLJ* deletion mutant has a slight growth defect. *B*. *dolosa* AU0158 or *fixLJ* were grown for 24 hours in LB in ambient oxygen with agitation (200rpm) or low oxygen (<5%) in a CampyGen Gas Generating System. CFU/mL was determined at indicated time point. Bars are means of a representative of three separate experiments with 3 biological replicates per experiment; error bars are S.D. * denotes P< 0.05 AU0158 vs *fixLJ* growth in ambient oxygen by t test. # denotes P< 0.05 AU0158 vs *fixLJ* growth in low oxygen by t test.
(TIFF)

S2 Fig Mice infected with *B*. *dolosa* show evidence of bacterial pneumonia. 7 days after infection with strain AU0158 (A&C) or the *fixLJ* deletion mutant (B&D), lung pathology was analyzed by H&E staining. Figures are representative from 2 separate experiments with 3–4 mice per group per experiment.
(TIF)

S3 Fig The *B*. *dolosa fixLJ* deletion mutant produces increased biofilm sensitive to proteinase K treatment. (A&B) Growth-adjusted biofilm formation of *B*. *dolosa* AU0158 constructs on PVC plates as measured by crystal violet staining at 48 hours. Strains were grown in TSB with 1% glucose at varying inocula. Biofilm staining was divided by O.D._600_ measured at 48 hour time point. Bars represent mean measurements of 5–6 replicates and error bars represent one standard deviation (representative of three independent experiments). *P<0.05 compared to AU0158 by 1-way ANOVA with Tukey’s multiple comparison test. (C) 48 hour AU0158 or *fixLJ* deletion mutant biofilm was treated with 120 u/mL DNase I, 3.18 mAu/mL proteinase K, or 50 μg/mL dispersin B for 24 hours at 37°C, when biofilm was measured by crystal violet staining. Bars represent mean measurements of 4–6 replicates and error bars represent one standard deviation (representative of two independent experiments). *P<0.05 compared to the corresponding untreated group by 1-way ANOVA with Dunnett’s multiple comparison test.
(TIFF)

S4 Fig The *B*. *dolosa fixLJ* deletion mutant has altered *fliC*, *flhD*, *flp*, and *fixK* expression. Relative expression of fliC (A&E), *flhD* (B&F), *flp* (C&G), *fixK* (D&H) in the *fixLJ* deletion mutant or complemented controls measured by qRT-PCR normalized to the expression of *gyrB* (A-D) or *rpoD* (E-H). Bars are means of 2–3 separate experiments with 2–3 biological replicates per experiment; error bars are S.D. *denotes P< 0.05 by t test.
(TIFF)

S5 Fig *B*. *dolosa* does not induce LDH release from A549 or THP-1 cells. A549 cells (A) or THP-1 cells treated with 200 nM PMA for 3 days (B&C) in a 24-well plate were infected with ~1x10^7^ CFU/well (MOI of ~100:1) of *B*. *dolosa* AU0158 or derivatives for 6 hours when LDH release was measured (A&B). (C) THP-1 derived macrophages were infected with *B*. *dolosa* AU0158 or derivatives for 2 hours when cells were washed and treated with media containing 1 mg/mL kanamycin for 24 hours when LDH release was measured. Percent cytotoxicity was determined based on LDH from cells treated with lysis buffer. Bars are means from a representative experiment from 3 separate experiments with 3–4 replicates per experiments; error bars depict S.D. There was no significant difference (*p* <0.05) by ANOVA with Tukey’s with multiple comparison test between AU0158 vs *fixLJ* deletion mutant, *ΔfixLJ* + *fixLJ*, *ΔfixLJ* + EV, or AU0158 vs *fliC* deletion.
(TIFF)

S1 Table Expression and fold change in *B*. *dolosa* AU0158 and *fixLJ* deletion mutant measured by RNA-seq. (XLSX)

S2 Table Oligonucleotides used for qRT-PCR. (DOCX)
