# Supplementary figures and images for: An Oxygen-Sensing Two-Component System in the Burkholderia cepacia Complex Regulates Biofilm, Intracellular Invasion, and Pathogenicity
Source: PLoS Pathog. 2017 Jan 3;13(1):e1006116. doi: 10.1371/journal.ppat.1006116 (PMC5234846; doi:10.1371/journal.ppat.1006116)

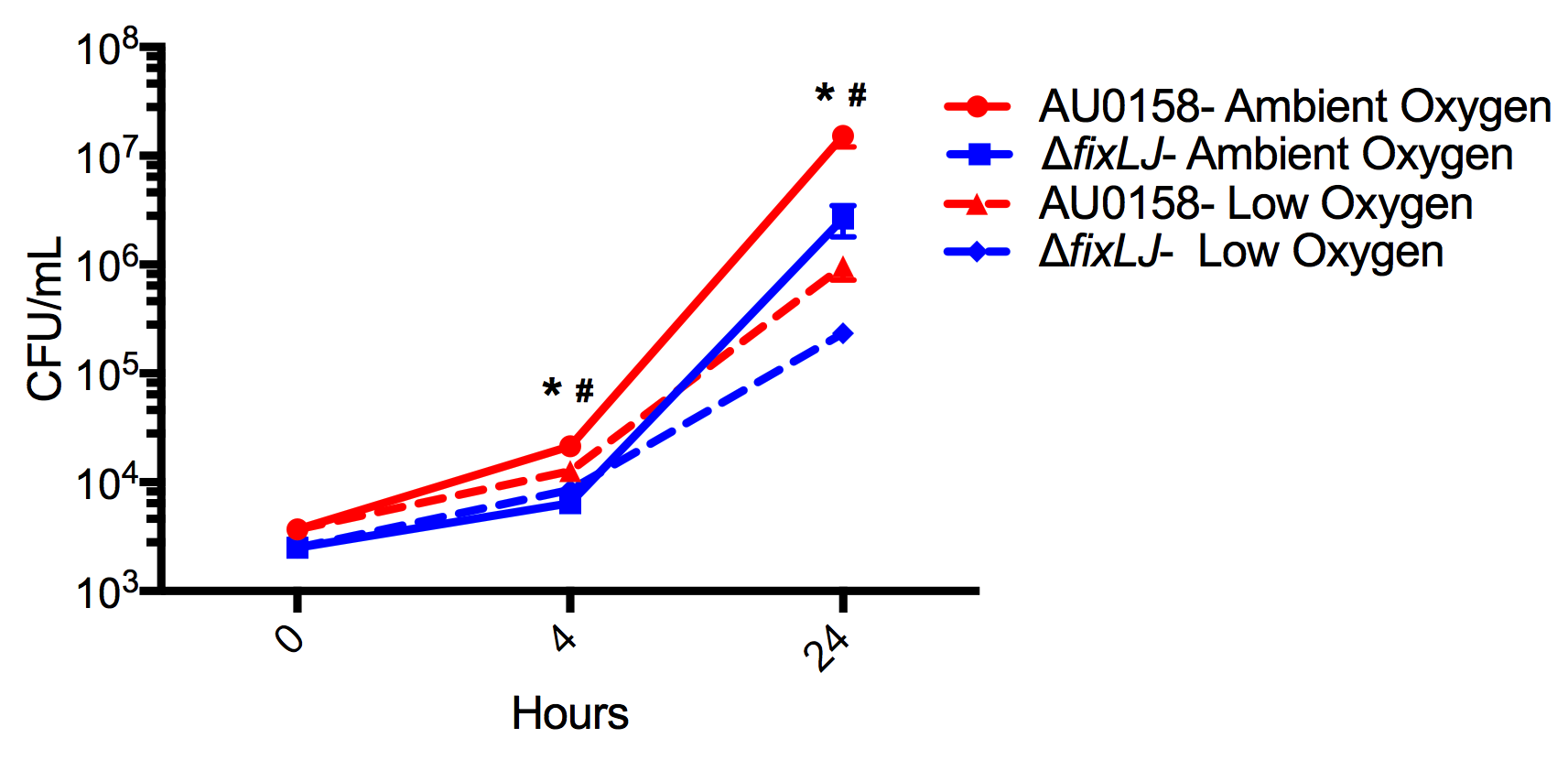

Supplement: S1 Fig — B. dolosa AU0158 or fixLJ were grown for 24 hours in LB in ambient oxygen with agitation (200rpm) or low oxygen (<5%) in a CampyGen Gas Generating System. CFU/mL was determined at indicated time point. Bars are means of a representative of three separate experiments with 3 biological replicates per experiment; error bars are S.D. * denotes P< 0.05 AU0158 vs fixLJ growth in ambient oxygen by t test. # denotes P< 0.05 AU0158 vs fixLJ growth in low oxygen by t test. (TIFF) [file ppat.1006116.s001.tiff]

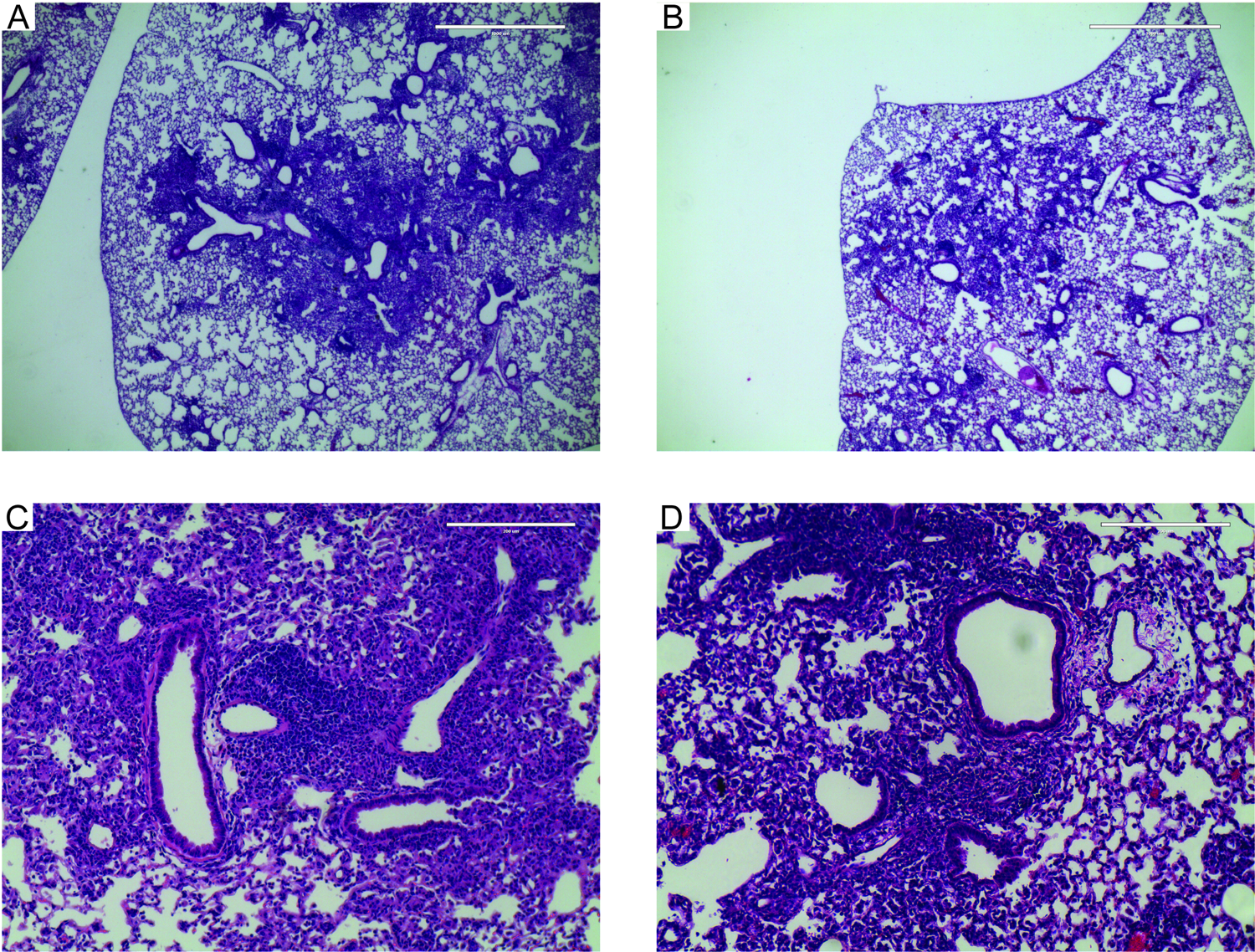

Supplement: S2 Fig — 7 days after infection with strain AU0158 (A&C) or the fixLJ deletion mutant (B&D), lung pathology was analyzed by H&E staining. Figures are representative from 2 separate experiments with 3–4 mice per group per experiment. (TIF) [file ppat.1006116.s002.tif]

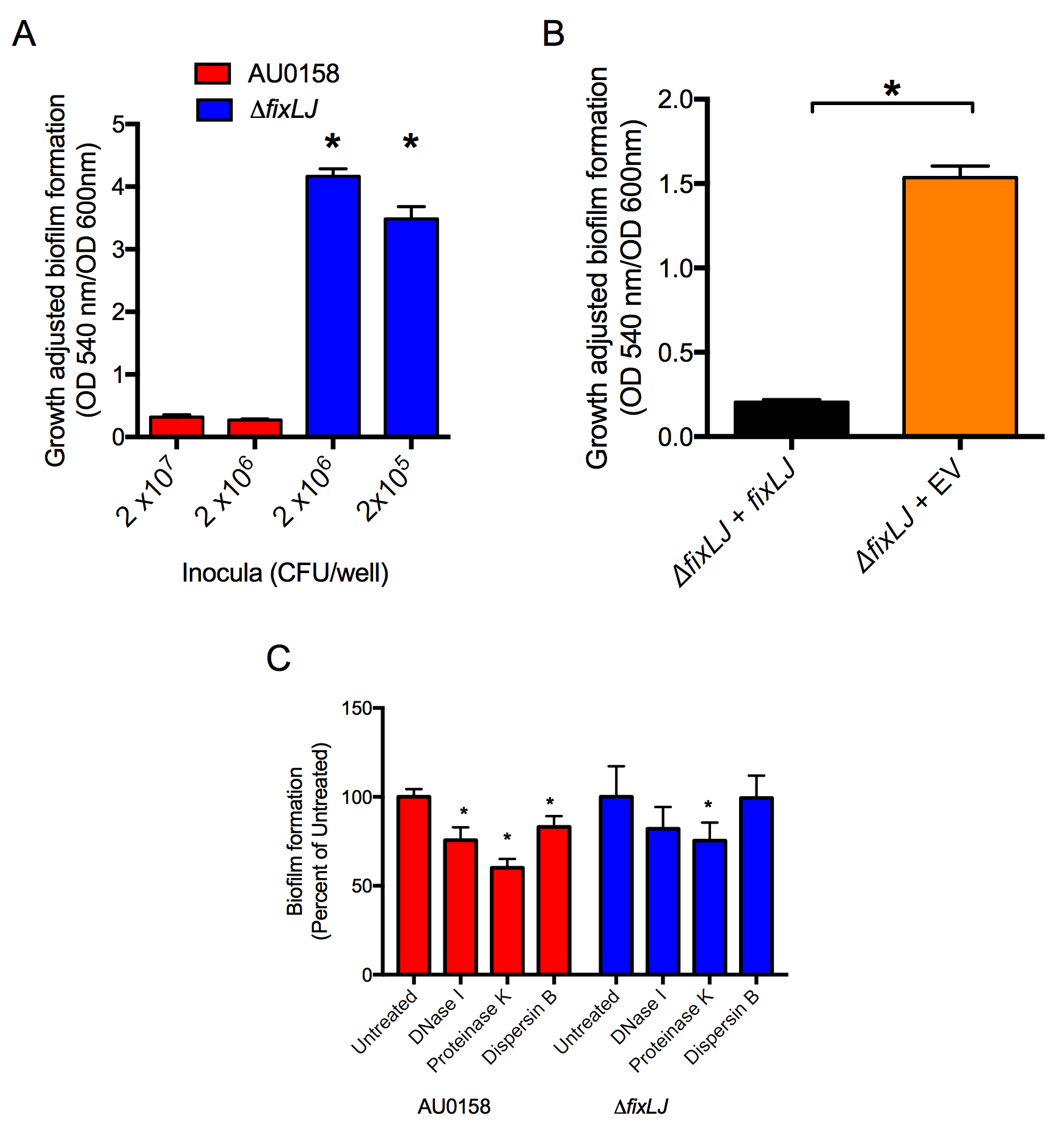

Supplement: S3 Fig — (A&B) Growth-adjusted biofilm formation of B. dolosa AU0158 constructs on PVC plates as measured by crystal violet staining at 48 hours. Strains were grown in TSB with 1% glucose at varying inocula. Biofilm staining was divided by O.D.600 measured at 48 hour time point. Bars represent mean measurements of 5–6 replicates and error bars represent one standard deviation (representative of three independent experiments). *P<0.05 compared to AU0158 by 1-way ANOVA with Tukey’s multiple comparison test. (C) 48 hour AU0158 or fixLJ deletion mutant biofilm was treated with 120 u/mL DNase I, 3.18 mAu/mL proteinase K, or 50 μg/mL dispersin B for 24 hours at 37°C, when biofilm was measured by crystal violet staining. Bars represent mean measurements of 4–6 replicates and error bars represent one standard deviation (representative of two independent experiments). *P<0.05 compared to the corresponding untreated group by 1-way ANOVA with Dunnett’s multiple comparison test. (TIFF) [file ppat.1006116.s003.tiff]

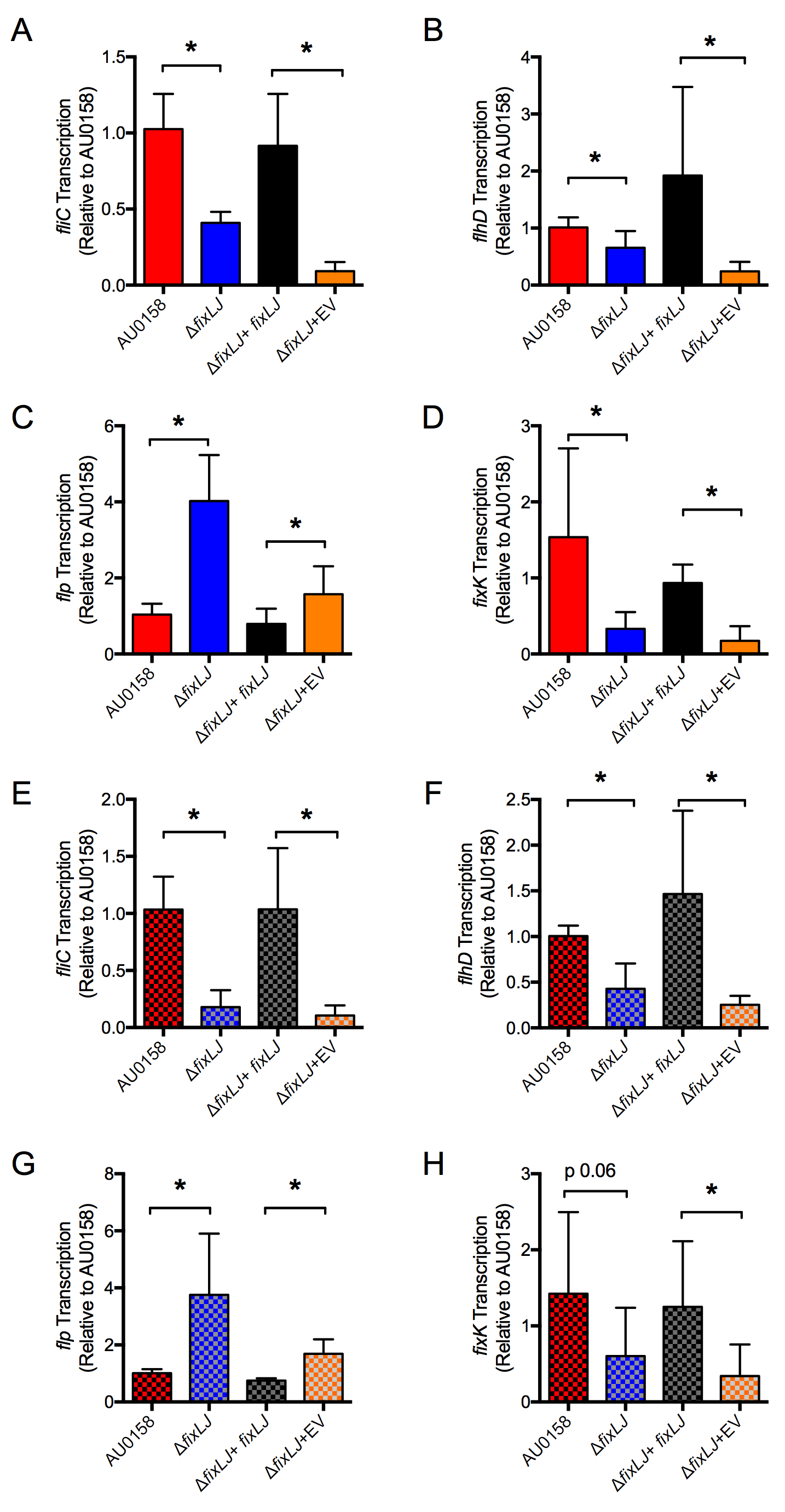

Supplement: S4 Fig — Relative expression of fliC (A&E), flhD (B&F), flp (C&G), fixK (D&H) in the fixLJ deletion mutant or complemented controls measured by qRT-PCR normalized to the expression of gyrB (A-D) or rpoD (E-H). Bars are means of 2–3 separate experiments with 2–3 biological replicates per experiment; error bars are S.D. *denotes P< 0.05 by t test. (TIFF) [file ppat.1006116.s004.tiff]

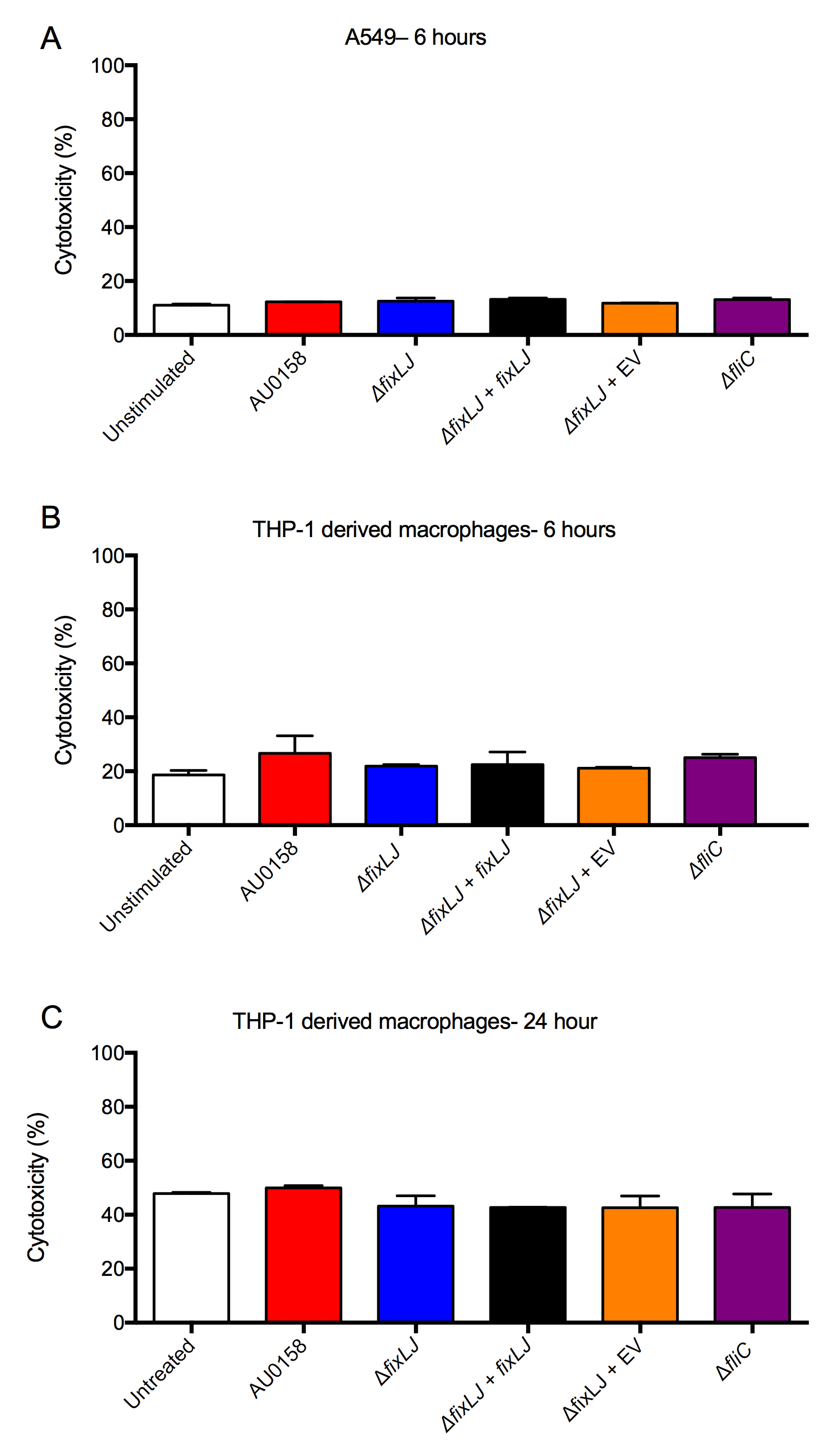

Supplement: S5 Fig — A549 cells (A) or THP-1 cells treated with 200 nM PMA for 3 days (B&C) in a 24-well plate were infected with ~1x10^7^ CFU/well (MOI of ~100:1) of B. dolosa AU0158 or derivatives for 6 hours when LDH release was measured (A&B). (C) THP-1 derived macrophages were infected with B. dolosa AU0158 or derivatives for 2 hours when cells were washed and treated with media containing 1 mg/mL kanamycin for 24 hours when LDH release was measured. Percent cytotoxicity was determined based on LDH from cells treated with lysis buffer. Bars are means from a representative experiment from 3 separate experiments with 3–4 replicates per experiments; error bars depict S.D. There was no significant difference (p <0.05) by ANOVA with Tukey’s with multiple comparison test between AU0158 vs fixLJ deletion mutant, ΔfixLJ + fixLJ, ΔfixLJ + EV, or AU0158 vs fliC deletion. (TIFF) [file ppat.1006116.s005.tiff]
