# Supplementary material for: An Oxygen-Sensing Two-Component System in the Burkholderia cepacia Complex Regulates Biofilm, Intracellular Invasion, and Pathogenicity
Source: PLoS Pathog. 2017 Jan 3;13(1):e1006116. doi: 10.1371/journal.ppat.1006116 (PMC5234846; doi:10.1371/journal.ppat.1006116)
Supplement: S2 Table — (DOCX) [file ppat.1006116.s007.docx]

**Table S2. Oligonucleotides used qRT-PCR**

| Gene |  | Sequence |
| --- | --- | --- |
| *gyrB*  (AK34_3072) | Forward | TTGCCGATAGAAGAACGTGAG |
|  | Reverse | GATCGGCAAGGACGATTACA |
| *rpoD* (AK34_4533) | Forward | TGATCCAGGAAGGCAACATC |
|  | Reverse | CGCGTAAGTCGAGAACTTGTA |
| *fliC* (AK34_2913) | Forward | AACGCAGACGTCGTTCAA |
|  | Reverse | GCCGAAGTCCACGGAAA |
| *flhD* (AK34_2903) | Forward | ATGCGGAACATGCCCATC |
|  | Reverse | GCGAAATGCTCAGTGAGATCAA |
| *flp* (AK34_1653) | Forward | CTGGAGGCCGACTTGAAA |
|  | Reverse | AACCTGGGCACCTGTATTT |
| *fixK* (AK34_4936) | Forward | CTACCTGAAGCGCGGTTATT |
|  | Reverse | ACCGTTTCGAGCGTCATC |
